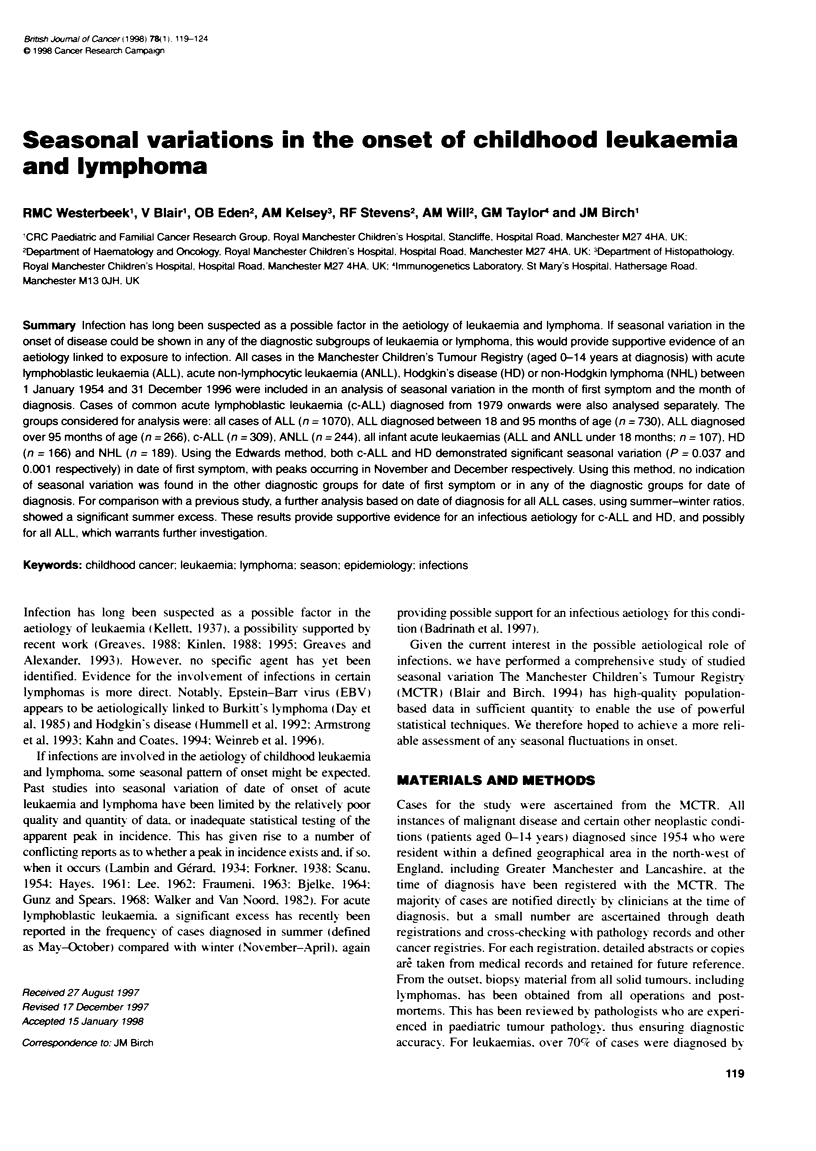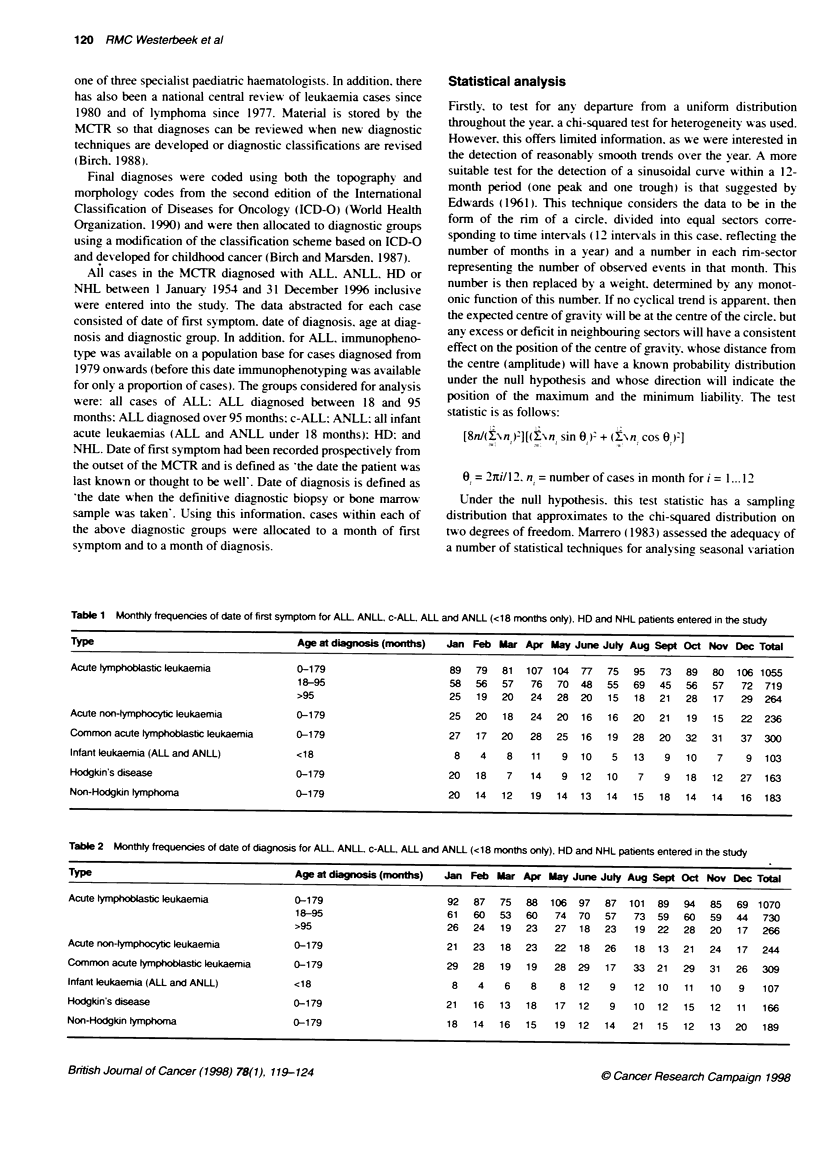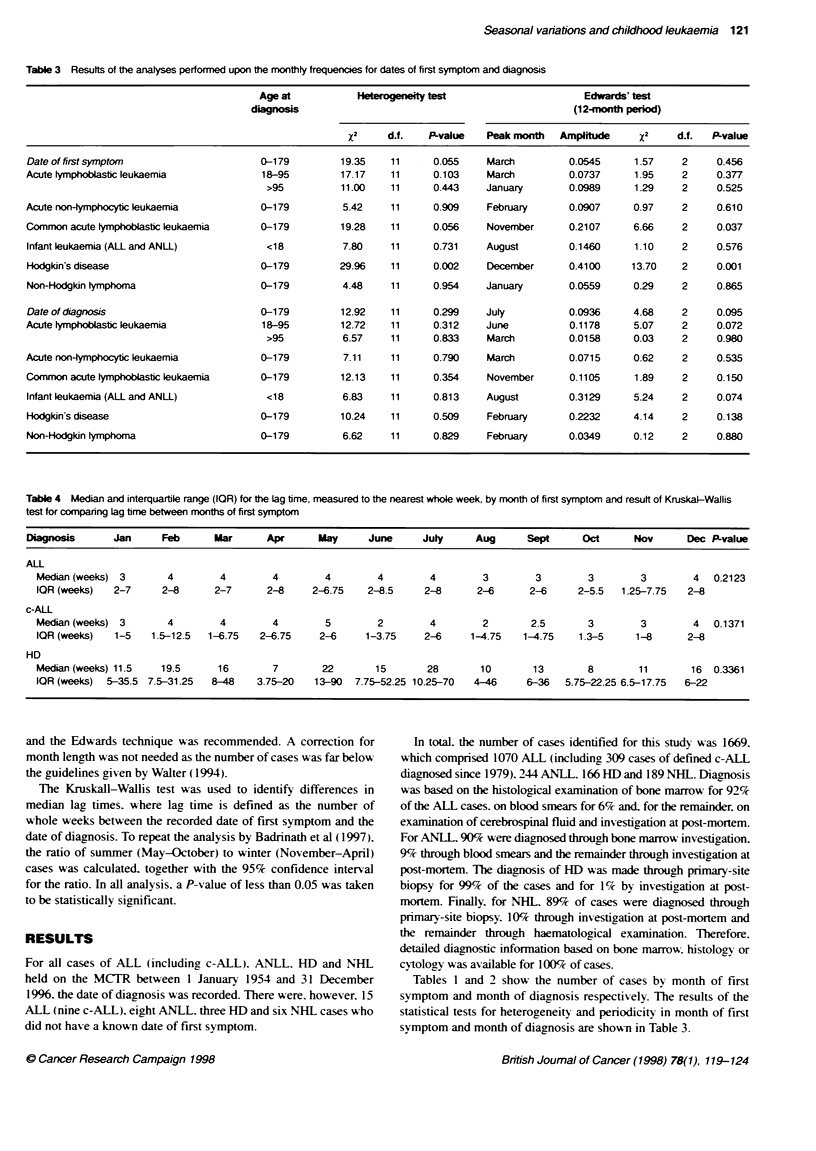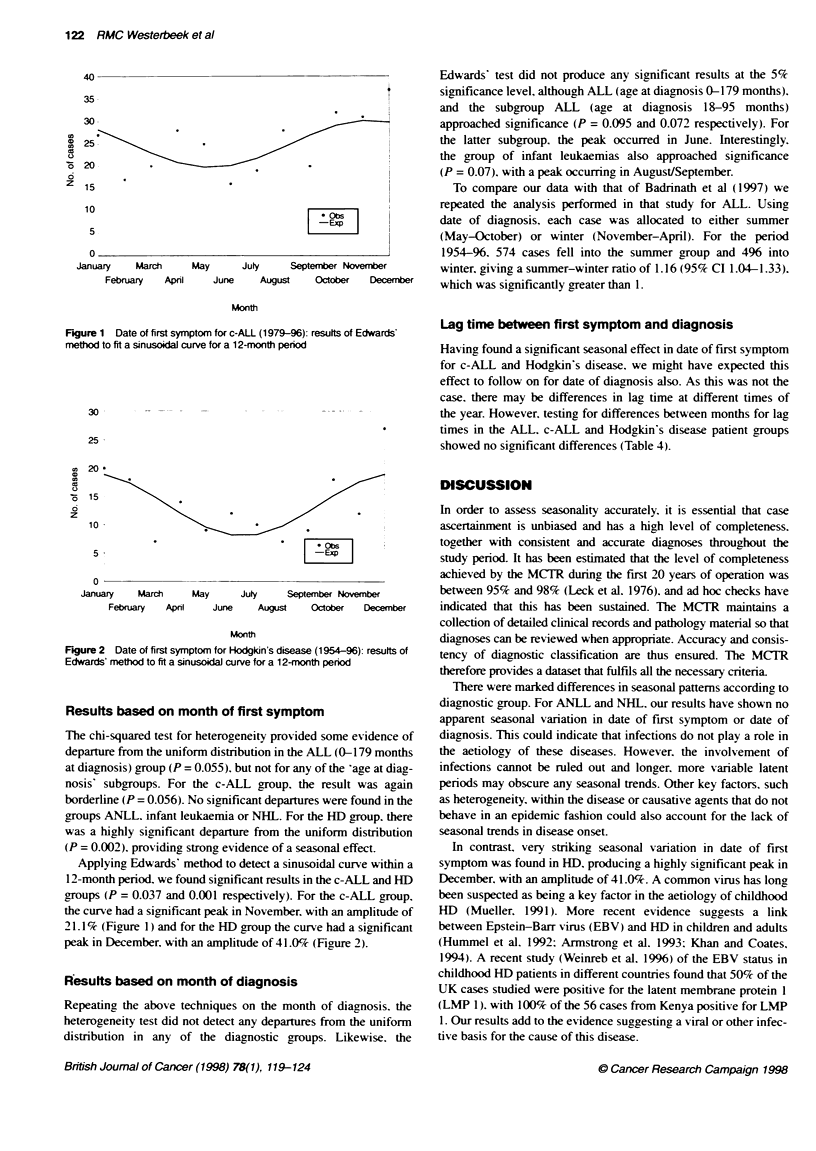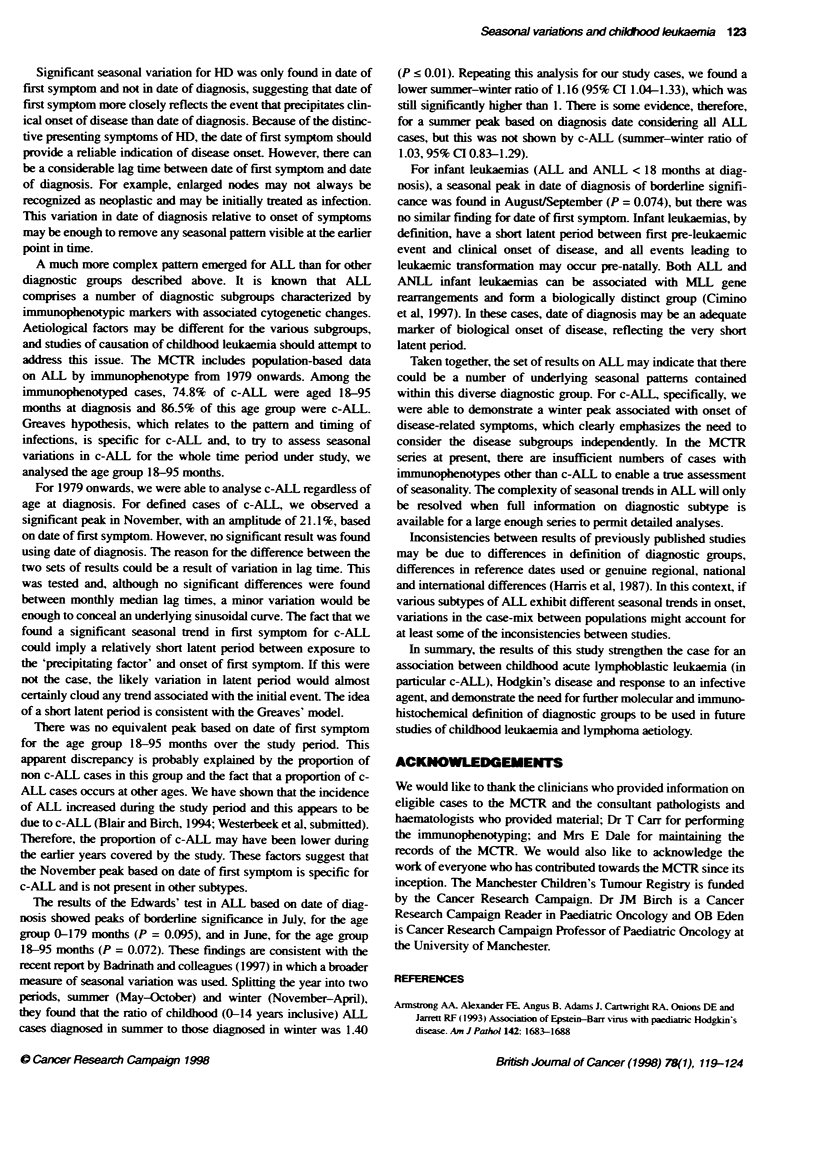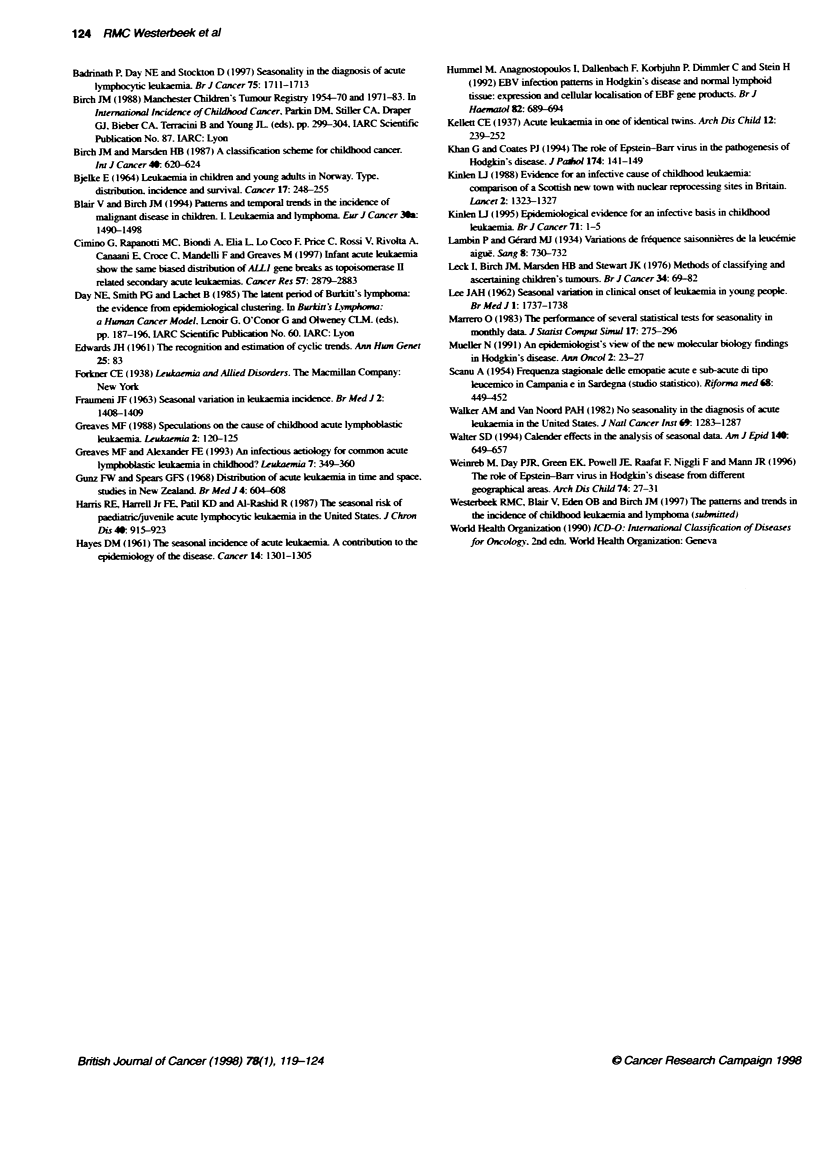# Seasonal variations in the onset of childhood leukaemia and lymphoma.

**DOI:** 10.1038/bjc.1998.452

**Published:** 1998-07

**Authors:** R. M. Westerbeek, V. Blair, O. B. Eden, A. M. Kelsey, R. F. Stevens, A. M. Will, G. M. Taylor, J. M. Birch

**Affiliations:** CRC Paediatric and Familial Cancer Research Group, Royal Manchester Children's Hospital, Stancliffe, UK.

## Abstract

Infection has long been suspected as a possible factor in the aetiology of leukaemia and lymphoma. If seasonal variation in the onset of disease could be shown in any of the diagnostic subgroups of leukaemia or lymphoma, this would provide supportive evidence of an aetiology linked to exposure to infection. All cases in the Manchester Children's Tumour Registry (aged 0-14 years at diagnosis) with acute lymphoblastic leukaemia (ALL), acute non-lymphocytic leukaemia (ANLL), Hodgkin's disease (HD) or non-Hodgkin lymphoma (NHL) between 1 January 1954 and 31 December 1996 were included in an analysis of seasonal variation in the month of first symptom and the month of diagnosis. Cases of common acute lymphoblastic leukaemia (c-ALL) diagnosed from 1979 onwards were also analysed separately. The groups considered for analysis were: all cases of ALL (n = 1070), ALL diagnosed between 18 and 95 months of age (n = 730), ALL diagnosed over 95 months of age (n = 266), c-ALL (n = 309), ANLL (n = 244), all infant acute leukaemias (ALL and ANLL under 18 months; n = 107), HD (n = 166) and NHL (n = 189). Using the Edwards method, both c-ALL and HD demonstrated significant seasonal variation (P = 0.037 and 0.001 respectively) in date of first symptom, with peaks occurring in November and December respectively. Using this method, no indication of seasonal variation was found in the other diagnostic groups for date of first symptom or in any of the diagnostic groups for date of diagnosis. For comparison with a previous study, a further analysis based on date of diagnosis for all ALL cases, using summer-winter ratios, showed a significant summer excess. These results provide supportive evidence for an infectious aetiology for c-ALL and HD, and possibly for all ALL, which warrants further investigation.


British Journal of Cancer (1998) 78(1). 119–124
© 1998 Cancer Research Campaign

### Seasonal variations in the onset of childhood leukaemia and lymphoma

**RMC Westerbeek[1], V Blair[1], OB Eden[2], AM Kelsey[3], RF Stevens[2], AM Will[2], GM Taylor[4] and JM Birch[1]**

[1]CRC Paediatric and Familial Cancer Research Group. Royal Manchester Children's Hospital. Standiffe. Hospital Road. Manchester M27 4HA. UK: [2]Department of Haematology and Oncology. Royal Manchester Children's Hospital. Hospital Road. Manchester M27 4HA. UK: [3]Department of Histopathology. Royal Manchester Children's Hospital. Hospital Road. Manchester M27 4HA. UK; [4]Immunogenetics Laboratory. St Mary's Hospital. Hathersage Road. Manchester M13 0JH. UK

**Summary** Infection has long been suspected as a possible factor in the aetiology of leukaemia and lymphoma. If seasonal variation in the onset of disease could be shown in any of the diagnostic subgroups of leukaemia or lymphoma, this would provide supportive evidence of an aetiology linked to exposure to infection. All cases in the Manchester Children's Tumour Registry (aged 0–14 years at diagnosis) with acute lymphoblastic leukaemia (ALL), acute non-lymphocytic leukaemia (ANLL), Hodgkin's disease (HD) or non-Hodgkin lymphoma (NHL) between 1 January 1954 and 31 December 1996 were included in an analysis of seasonal variation in the month of first symptom and the month of diagnosis. Cases of common acute lymphoblastic leukaemia (c-ALL) diagnosed from 1979 onwards were also analysed separately. The groups considered for analysis were: all cases of ALL ($n = 1070$), ALL diagnosed between 18 and 95 months of age ($n = 730$), ALL diagnosed over 95 months of age ($n = 266$), c-ALL ($n = 309$), ANLL ($n = 244$), all infant acute leukaemias (ALL and ANLL under 18 months; $n = 107$), HD ($n = 166$) and NHL ($n = 189$). Using the Edwards method, both c-ALL and HD demonstrated significant seasonal variation ($P = 0.037$ and 0.001 respectively) in date of first symptom, with peaks occurring in November and December respectively. Using this method, no indication of seasonal variation was found in the other diagnostic groups for date of first symptom or in any of the diagnostic groups for date of diagnosis. For comparison with a previous study, a further analysis based on date of diagnosis for all ALL cases, using summer–winter ratios, showed a significant summer excess. These results provide supportive evidence for an infectious aetiology for c-ALL and HD, and possibly for all ALL, which warrants further investigation.

**Keywords:** childhood cancer; leukaemia; lymphoma; season; epidemiology; infections

Infection has long been suspected as a possible factor in the aetiology of leukaemia (Kellett. 1937). a possibility supported by recent work (Greaves. 1988: Kinlen. 1988: 1995: Greaves and Alexander. 1993). However. no specific agent has yet been identified. Evidence for the involvement of infections in certain lymphomas is more direct. Notably. Epstein–Barr virus (EBV) appears to be aetiologically linked to Burkitt's lymphoma (Day et al. 1985) and Hodgkin's disease (Hummell et al. 1992: Armstrong et al. 1993: Kahn and Coates. 1994: Weinreb et al. 1996).

If infections are involved in the aetiology of childhood leukaemia and lymphoma. some seasonal pattern of onset might be expected. Past studies into seasonal variation of date of onset of acute leukaemia and lymphoma have been limited by the relatively poor quality and quantity of data. or inadequate statistical testing of the apparent peak in incidence. This has given rise to a number of conflicting reports as to whether a peak in incidence exists and. if so. when it occurs (Lambin and Gérard. 1934: Forkner. 1938: Scanu. 1954: Hayes. 1961: Lee. 1962: Fraumeni. 1963: Bjelke. 1964: Gunz and Spears. 1968: Walker and Van Noord. 1982). For acute lymphoblastic leukaemia. a significant excess has recently been reported in the frequency of cases diagnosed in summer (defined as May–October) compared with winter (November–April). again providing possible support for an infectious aetiology for this condition (Badrinath et al. 1997).

Given the current interest in the possible aetiological role of infections. we have performed a comprehensive study of studied seasonal variation The Manchester Children's Tumour Registry (MCTR) (Blair and Birch. 1994) has high-quality population-based data in sufficient quantity to enable the use of powerful statistical techniques. We therefore hoped to achieve a more reliable assessment of any seasonal fluctuations in onset.

### MATERIALS AND METHODS

Cases for the study were ascertained from the MCTR. All instances of malignant disease and certain other neoplastic conditions (patients aged 0–14 years) diagnosed since 1954 who were resident within a defined geographical area in the north-west of England. including Greater Manchester and Lancashire. at the time of diagnosis have been registered with the MCTR. The majority of cases are notified directly by clinicians at the time of diagnosis. but a small number are ascertained through death registrations and cross-checking with pathology records and other cancer registries. For each registration. detailed abstracts or copies are taken from medical records and retained for future reference. From the outset. biopsy material from all solid tumours. including lymphomas. has been obtained from all operations and post-mortems. This has been reviewed by pathologists who are experienced in paediatric tumour pathology. thus ensuring diagnostic accuracy. For leukaemias. over 70% of cases were diagnosed by

Received 27 August 1997
Revised 17 December 1997
Accepted 15 January 1998

Correspondence to: JM Birch one of three specialist paediatric haematologists. In addition, there has also been a national central review of leukaemia cases since 1980 and of lymphoma since 1977. Material is stored by the MCTR so that diagnoses can be reviewed when new diagnostic techniques are developed or diagnostic classifications are revised (Birch, 1988).

Final diagnoses were coded using both the topography and morphology codes from the second edition of the International Classification of Diseases for Oncology (ICD-O) (World Health Organization, 1990) and were then allocated to diagnostic groups using a modification of the classification scheme based on ICD-O and developed for childhood cancer (Birch and Marsden, 1987).

All cases in the MCTR diagnosed with ALL, ANLL, HD or NHL between 1 January 1954 and 31 December 1996 inclusive were entered into the study. The data abstracted for each case consisted of date of first symptom, date of diagnosis, age at diagnosis and diagnostic group. In addition, for ALL, immunophenotype was available on a population base for cases diagnosed from 1979 onwards (before this date immunophenotyping was available for only a proportion of cases). The groups considered for analysis were: all cases of ALL; ALL diagnosed between 18 and 95 months; ALL diagnosed over 95 months; c-ALL; ANLL; all infant acute leukaemias (ALL and ANLL under 18 months); HD; and NHL. Date of first symptom had been recorded prospectively from the outset of the MCTR and is defined as 'the date the patient was last known or thought to be well'. Date of diagnosis is defined as 'the date when the definitive diagnostic biopsy or bone marrow sample was taken'. Using this information, cases within each of the above diagnostic groups were allocated to a month of first symptom and to a month of diagnosis.

### Statistical analysis

Firstly, to test for any departure from a uniform distribution throughout the year, a chi-squared test for heterogeneity was used. However, this offers limited information, as we were interested in the detection of reasonably smooth trends over the year. A more suitable test for the detection of a sinusoidal curve within a 12-month period (one peak and one trough) is that suggested by Edwards (1961). This technique considers the data to be in the form of the rim of a circle, divided into equal sectors corresponding to time intervals (12 intervals in this case, reflecting the number of months in a year) and a number in each rim-sector representing the number of observed events in that month. This number is then replaced by a weight, determined by any monotonic function of this number. If no cyclical trend is apparent, then the expected centre of gravity will be at the centre of the circle, but any excess or deficit in neighbouring sectors will have a consistent effect on the position of the centre of gravity, whose distance from the centre (amplitude) will have a known probability distribution under the null hypothesis and whose direction will indicate the position of the maximum and the minimum liability. The test statistic is as follows:

$$[8n/(\sum_{i=1}^{12}\sqrt{n_i})^2][(\sum_{i=1}^{12}\sqrt{n_i}\sin\theta_i)^2 + (\sum_{i=1}^{12}\sqrt{n_i}\cos\theta_i)^2]$$

$\theta_i = 2\pi i/12$, $n_i$ = number of cases in month for $i = 1...12$

Under the null hypothesis, this test statistic has a sampling distribution that approximates to the chi-squared distribution on two degrees of freedom. Marrero (1983) assessed the adequacy of a number of statistical techniques for analysing seasonal variation

**Table 1** Monthly frequencies of date of first symptom for ALL, ANLL, c-ALL, ALL and ANLL (<18 months only), HD and NHL patients entered in the study

| Type | Age at diagnosis (months) | Jan | Feb | Mar | Apr | May | June | July | Aug | Sept | Oct | Nov | Dec | Total |
|---|---|---|---|---|---|---|---|---|---|---|---|---|---|---|
| Acute lymphoblastic leukaemia | 0–179 | 89 | 79 | 81 | 107 | 104 | 77 | 75 | 95 | 73 | 89 | 80 | 106 | 1055 |
| | 18–95 | 58 | 56 | 57 | 76 | 70 | 48 | 55 | 69 | 45 | 56 | 57 | 72 | 719 |
| | >95 | 25 | 19 | 20 | 24 | 28 | 20 | 15 | 18 | 21 | 28 | 17 | 29 | 264 |
| Acute non-lymphocytic leukaemia | 0–179 | 25 | 20 | 18 | 24 | 20 | 16 | 16 | 20 | 21 | 19 | 15 | 22 | 236 |
| Common acute lymphoblastic leukaemia | 0–179 | 27 | 17 | 20 | 28 | 25 | 16 | 19 | 28 | 20 | 32 | 31 | 37 | 300 |
| Infant leukaemia (ALL and ANLL) | <18 | 8 | 4 | 8 | 11 | 9 | 10 | 5 | 13 | 9 | 10 | 7 | 9 | 103 |
| Hodgkin's disease | 0–179 | 20 | 18 | 7 | 14 | 9 | 12 | 10 | 7 | 9 | 18 | 12 | 27 | 163 |
| Non-Hodgkin lymphoma | 0–179 | 20 | 14 | 12 | 19 | 14 | 13 | 14 | 15 | 18 | 14 | 14 | 16 | 183 |

**Table 2** Monthly frequencies of date of diagnosis for ALL, ANLL, c-ALL, ALL and ANLL (<18 months only), HD and NHL patients entered in the study

| Type | Age at diagnosis (months) | Jan | Feb | Mar | Apr | May | June | July | Aug | Sept | Oct | Nov | Dec | Total |
|---|---|---|---|---|---|---|---|---|---|---|---|---|---|---|
| Acute lymphoblastic leukaemia | 0–179 | 92 | 87 | 75 | 88 | 106 | 97 | 87 | 101 | 89 | 94 | 85 | 69 | 1070 |
| | 18–95 | 61 | 60 | 53 | 60 | 74 | 70 | 57 | 73 | 59 | 60 | 59 | 44 | 730 |
| | >95 | 26 | 24 | 19 | 23 | 27 | 18 | 23 | 19 | 22 | 28 | 20 | 17 | 266 |
| Acute non-lymphocytic leukaemia | 0–179 | 21 | 23 | 18 | 23 | 22 | 18 | 26 | 18 | 13 | 21 | 24 | 17 | 244 |
| Common acute lymphoblastic leukaemia | 0–179 | 29 | 28 | 19 | 19 | 28 | 29 | 17 | 33 | 21 | 29 | 31 | 26 | 309 |
| Infant leukaemia (ALL and ANLL) | <18 | 8 | 4 | 6 | 8 | 8 | 12 | 9 | 12 | 10 | 11 | 10 | 9 | 107 |
| Hodgkin's disease | 0–179 | 21 | 16 | 13 | 18 | 17 | 12 | 9 | 10 | 12 | 15 | 12 | 11 | 166 |
| Non-Hodgkin lymphoma | 0–179 | 18 | 14 | 16 | 15 | 19 | 12 | 14 | 21 | 15 | 12 | 13 | 20 | 189 |

*© Cancer Research Campaign 1998*

**Table 3** Results of the analyses performed upon the monthly frequencies for dates of first symptom and diagnosis

| | Age at diagnosis | Heterogeneity test | | | Edwards' test (12-month period) | | | | |
|---|---|---|---|---|---|---|---|---|---|
| | | $\chi^2$ | d.f. | *P*-value | Peak month | Amplitude | $\chi^2$ | d.f. | *P*-value |
| *Date of first symptom* | 0–179 | 19.35 | 11 | 0.055 | March | 0.0545 | 1.57 | 2 | 0.456 |
| Acute lymphoblastic leukaemia | 18–95 | 17.17 | 11 | 0.103 | March | 0.0737 | 1.95 | 2 | 0.377 |
| | >95 | 11.00 | 11 | 0.443 | January | 0.0989 | 1.29 | 2 | 0.525 |
| Acute non-lymphocytic leukaemia | 0–179 | 5.42 | 11 | 0.909 | February | 0.0907 | 0.97 | 2 | 0.610 |
| Common acute lymphoblastic leukaemia | 0–179 | 19.28 | 11 | 0.056 | November | 0.2107 | 6.66 | 2 | 0.037 |
| Infant leukaemia (ALL and ANLL) | <18 | 7.80 | 11 | 0.731 | August | 0.1460 | 1.10 | 2 | 0.576 |
| Hodgkin's disease | 0–179 | 29.96 | 11 | 0.002 | December | 0.4100 | 13.70 | 2 | 0.001 |
| Non-Hodgkin lymphoma | 0–179 | 4.48 | 11 | 0.954 | January | 0.0559 | 0.29 | 2 | 0.865 |
| *Date of diagnosis* | 0–179 | 12.92 | 11 | 0.299 | July | 0.0936 | 4.68 | 2 | 0.095 |
| Acute lymphoblastic leukaemia | 18–95 | 12.72 | 11 | 0.312 | June | 0.1178 | 5.07 | 2 | 0.072 |
| | >95 | 6.57 | 11 | 0.833 | March | 0.0158 | 0.03 | 2 | 0.980 |
| Acute non-lymphocytic leukaemia | 0–179 | 7.11 | 11 | 0.790 | March | 0.0715 | 0.62 | 2 | 0.535 |
| Common acute lymphoblastic leukaemia | 0–179 | 12.13 | 11 | 0.354 | November | 0.1105 | 1.89 | 2 | 0.150 |
| Infant leukaemia (ALL and ANLL) | <18 | 6.83 | 11 | 0.813 | August | 0.3129 | 5.24 | 2 | 0.074 |
| Hodgkin's disease | 0–179 | 10.24 | 11 | 0.509 | February | 0.2232 | 4.14 | 2 | 0.138 |
| Non-Hodgkin lymphoma | 0–179 | 6.62 | 11 | 0.829 | February | 0.0349 | 0.12 | 2 | 0.880 |

**Table 4** Median and interquartile range (IQR) for the lag time, measured to the nearest whole week, by month of first symptom and result of Kruskal–Wallis test for comparing lag time between months of first symptom

| Diagnosis | Jan | Feb | Mar | Apr | May | June | July | Aug | Sept | Oct | Nov | Dec | *P*-value |
|---|---|---|---|---|---|---|---|---|---|---|---|---|---|
| ALL | | | | | | | | | | | | | |
| Median (weeks) | 3 | 4 | 4 | 4 | 4 | 4 | 4 | 3 | 3 | 3 | 3 | 4 | 0.2123 |
| IQR (weeks) | 2–7 | 2–8 | 2–7 | 2–8 | 2–6.75 | 2–8.5 | 2–8 | 2–6 | 2–6 | 2–5.5 | 1.25–7.75 | 2–8 | |
| c-ALL | | | | | | | | | | | | | |
| Median (weeks) | 3 | 4 | 4 | 4 | 5 | 2 | 4 | 2 | 2.5 | 3 | 3 | 4 | 0.1371 |
| IQR (weeks) | 1–5 | 1.5–12.5 | 1–6.75 | 2–6.75 | 2–6 | 1–3.75 | 2–6 | 1–4.75 | 1–4.75 | 1.3–5 | 1–8 | 2–8 | |
| HD | | | | | | | | | | | | | |
| Median (weeks) | 11.5 | 19.5 | 16 | 7 | 22 | 15 | 28 | 10 | 13 | 8 | 11 | 16 | 0.3361 |
| IQR (weeks) | 5–35.5 | 7.5–31.25 | 8–48 | 3.75–20 | 13–90 | 7.75–52.25 | 10.25–70 | 4–46 | 6–36 | 5.75–22.25 | 6.5–17.75 | 6–22 | |

and the Edwards technique was recommended. A correction for month length was not needed as the number of cases was far below the guidelines given by Walter (1994).

The Kruskall–Wallis test was used to identify differences in median lag times, where lag time is defined as the number of whole weeks between the recorded date of first symptom and the date of diagnosis. To repeat the analysis by Badrinath et al (1997), the ratio of summer (May–October) to winter (November–April) cases was calculated, together with the 95% confidence interval for the ratio. In all analysis, a *P*-value of less than 0.05 was taken to be statistically significant.

### RESULTS

For all cases of ALL (including c-ALL), ANLL, HD and NHL held on the MCTR between 1 January 1954 and 31 December 1996, the date of diagnosis was recorded. There were, however, 15 ALL (nine c-ALL), eight ANLL, three HD and six NHL cases who did not have a known date of first symptom.

In total, the number of cases identified for this study was 1669, which comprised 1070 ALL (including 309 cases of defined c-ALL diagnosed since 1979), 244 ANLL, 166 HD and 189 NHL. Diagnosis was based on the histological examination of bone marrow for 92% of the ALL cases, on blood smears for 6% and, for the remainder, on examination of cerebrospinal fluid and investigation at post-mortem. For ANLL, 90% were diagnosed through bone marrow investigation, 9% through blood smears and the remainder through investigation at post-mortem. The diagnosis of HD was made through primary-site biopsy for 99% of the cases and for 1% by investigation at post-mortem. Finally, for NHL, 89% of cases were diagnosed through primary-site biopsy, 10% through investigation at post-mortem and the remainder through haematological examination. Therefore, detailed diagnostic information based on bone marrow, histology or cytology was available for 100% of cases.

Tables 1 and 2 show the number of cases by month of first symptom and month of diagnosis respectively. The results of the statistical tests for heterogeneity and periodicity in month of first symptom and month of diagnosis are shown in Table 3.

© Cancer Research Campaign 1998

**Figure 1** Date of first symptom for c-ALL (1979–96): results of Edwards' method to fit a sinusoidal curve for a 12-month period

**Figure 2** Date of first symptom for Hodgkin's disease (1954–96): results of Edwards' method to fit a sinusoidal curve for a 12-month period

### Results based on month of first symptom

The chi-squared test for heterogeneity provided some evidence of departure from the uniform distribution in the ALL (0–179 months at diagnosis) group ($P = 0.055$), but not for any of the 'age at diagnosis' subgroups. For the c-ALL group, the result was again borderline ($P = 0.056$). No significant departures were found in the groups ANLL, infant leukaemia or NHL. For the HD group, there was a highly significant departure from the uniform distribution ($P = 0.002$), providing strong evidence of a seasonal effect.

Applying Edwards' method to detect a sinusoidal curve within a 12-month period, we found significant results in the c-ALL and HD groups ($P = 0.037$ and 0.001 respectively). For the c-ALL group, the curve had a significant peak in November, with an amplitude of 21.1% (Figure 1) and for the HD group the curve had a significant peak in December, with an amplitude of 41.0% (Figure 2).

### Results based on month of diagnosis

Repeating the above techniques on the month of diagnosis, the heterogeneity test did not detect any departures from the uniform distribution in any of the diagnostic groups. Likewise, the Edwards' test did not produce any significant results at the 5% significance level, although ALL (age at diagnosis 0–179 months), and the subgroup ALL (age at diagnosis 18–95 months) approached significance ($P = 0.095$ and 0.072 respectively). For the latter subgroup, the peak occurred in June. Interestingly, the group of infant leukaemias also approached significance ($P = 0.07$), with a peak occurring in August/September.

To compare our data with that of Badrinath et al (1997) we repeated the analysis performed in that study for ALL. Using date of diagnosis, each case was allocated to either summer (May–October) or winter (November–April). For the period 1954–96, 574 cases fell into the summer group and 496 into winter, giving a summer–winter ratio of 1.16 (95% CI 1.04–1.33), which was significantly greater than 1.

### Lag time between first symptom and diagnosis

Having found a significant seasonal effect in date of first symptom for c-ALL and Hodgkin's disease, we might have expected this effect to follow on for date of diagnosis also. As this was not the case, there may be differences in lag time at different times of the year. However, testing for differences between months for lag times in the ALL, c-ALL and Hodgkin's disease patient groups showed no significant differences (Table 4).

### DISCUSSION

In order to assess seasonality accurately, it is essential that case ascertainment is unbiased and has a high level of completeness, together with consistent and accurate diagnoses throughout the study period. It has been estimated that the level of completeness achieved by the MCTR during the first 20 years of operation was between 95% and 98% (Leck et al, 1976), and ad hoc checks have indicated that this has been sustained. The MCTR maintains a collection of detailed clinical records and pathology material so that diagnoses can be reviewed when appropriate. Accuracy and consistency of diagnostic classification are thus ensured. The MCTR therefore provides a dataset that fulfils all the necessary criteria.

There were marked differences in seasonal patterns according to diagnostic group. For ANLL and NHL, our results have shown no apparent seasonal variation in date of first symptom or date of diagnosis. This could indicate that infections do not play a role in the aetiology of these diseases. However, the involvement of infections cannot be ruled out and longer, more variable latent periods may obscure any seasonal trends. Other key factors, such as heterogeneity, within the disease or causative agents that do not behave in an epidemic fashion could also account for the lack of seasonal trends in disease onset.

In contrast, very striking seasonal variation in date of first symptom was found in HD, producing a highly significant peak in December, with an amplitude of 41.0%. A common virus has long been suspected as being a key factor in the aetiology of childhood HD (Mueller, 1991). More recent evidence suggests a link between Epstein–Barr virus (EBV) and HD in children and adults (Hummel et al, 1992; Armstrong et al, 1993; Khan and Coates, 1994). A recent study (Weinreb et al, 1996) of the EBV status in childhood HD patients in different countries found that 50% of the UK cases studied were positive for the latent membrane protein 1 (LMP 1), with 100% of the 56 cases from Kenya positive for LMP 1. Our results add to the evidence suggesting a viral or other infective basis for the cause of this disease.

© Cancer Research Campaign 1998

Significant seasonal variation for HD was only found in date of first symptom and not in date of diagnosis, suggesting that date of first symptom more closely reflects the event that precipitates clinical onset of disease than date of diagnosis. Because of the distinctive presenting symptoms of HD, the date of first symptom should provide a reliable indication of disease onset. However, there can be a considerable lag time between date of first symptom and date of diagnosis. For example, enlarged nodes may not always be recognized as neoplastic and may be initially treated as infection. This variation in date of diagnosis relative to onset of symptoms may be enough to remove any seasonal pattern visible at the earlier point in time.

A much more complex pattern emerged for ALL than for other diagnostic groups described above. It is known that ALL comprises a number of diagnostic subgroups characterized by immunophenotypic markers with associated cytogenetic changes. Aetiological factors may be different for the various subgroups, and studies of causation of childhood leukaemia should attempt to address this issue. The MCTR includes population-based data on ALL by immunophenotype from 1979 onwards. Among the immunophenotyped cases, 74.8% of c-ALL were aged 18–95 months at diagnosis and 86.5% of this age group were c-ALL. Greaves hypothesis, which relates to the pattern and timing of infections, is specific for c-ALL and, to try to assess seasonal variations in c-ALL for the whole time period under study, we analysed the age group 18–95 months.

For 1979 onwards, we were able to analyse c-ALL regardless of age at diagnosis. For defined cases of c-ALL, we observed a significant peak in November, with an amplitude of 21.1%, based on date of first symptom. However, no significant result was found using date of diagnosis. The reason for the difference between the two sets of results could be a result of variation in lag time. This was tested and, although no significant differences were found between monthly median lag times, a minor variation would be enough to conceal an underlying sinusoidal curve. The fact that we found a significant seasonal trend in first symptom for c-ALL could imply a relatively short latent period between exposure to the 'precipitating factor' and onset of first symptom. If this were not the case, the likely variation in latent period would almost certainly cloud any trend associated with the initial event. The idea of a short latent period is consistent with the Greaves' model.

There was no equivalent peak based on date of first symptom for the age group 18–95 months over the study period. This apparent discrepancy is probably explained by the proportion of non c-ALL cases in this group and the fact that a proportion of c-ALL cases occurs at other ages. We have shown that the incidence of ALL increased during the study period and this appears to be due to c-ALL (Blair and Birch, 1994; Westerbeek et al, submitted). Therefore, the proportion of c-ALL may have been lower during the earlier years covered by the study. These factors suggest that the November peak based on date of first symptom is specific for c-ALL and is not present in other subtypes.

The results of the Edwards' test in ALL based on date of diagnosis showed peaks of borderline significance in July, for the age group 0–179 months ($P = 0.095$), and in June, for the age group 18–95 months ($P = 0.072$). These findings are consistent with the recent report by Badrinath and colleagues (1997) in which a broader measure of seasonal variation was used. Splitting the year into two periods, summer (May–October) and winter (November–April), they found that the ratio of childhood (0–14 years inclusive) ALL cases diagnosed in summer to those diagnosed in winter was 1.40 ($P \leq 0.01$). Repeating this analysis for our study cases, we found a lower summer–winter ratio of 1.16 (95% CI 1.04–1.33), which was still significantly higher than 1. There is some evidence, therefore, for a summer peak based on diagnosis date considering all ALL cases, but this was not shown by c-ALL (summer–winter ratio of 1.03, 95% CI 0.83–1.29).

For infant leukaemias (ALL and ANLL < 18 months at diagnosis), a seasonal peak in date of diagnosis of borderline significance was found in August/September ($P = 0.074$), but there was no similar finding for date of first symptom. Infant leukaemias, by definition, have a short latent period between first pre-leukaemic event and clinical onset of disease, and all events leading to leukaemic transformation may occur pre-natally. Both ALL and ANLL infant leukaemias can be associated with MLL gene rearrangements and form a biologically distinct group (Cimino et al, 1997). In these cases, date of diagnosis may be an adequate marker of biological onset of disease, reflecting the very short latent period.

Taken together, the set of results on ALL may indicate that there could be a number of underlying seasonal patterns contained within this diverse diagnostic group. For c-ALL, specifically, we were able to demonstrate a winter peak associated with onset of disease-related symptoms, which clearly emphasizes the need to consider the disease subgroups independently. In the MCTR series at present, there are insufficient numbers of cases with immunophenotypes other than c-ALL to enable a true assessment of seasonality. The complexity of seasonal trends in ALL will only be resolved when full information on diagnostic subtype is available for a large enough series to permit detailed analyses.

Inconsistencies between results of previously published studies may be due to differences in definition of diagnostic groups, differences in reference dates used or genuine regional, national and international differences (Harris et al, 1987). In this context, if various subtypes of ALL exhibit different seasonal trends in onset, variations in the case-mix between populations might account for at least some of the inconsistencies between studies.

In summary, the results of this study strengthen the case for an association between childhood acute lymphoblastic leukaemia (in particular c-ALL), Hodgkin's disease and response to an infective agent, and demonstrate the need for further molecular and immunohistochemical definition of diagnostic groups to be used in future studies of childhood leukaemia and lymphoma aetiology.

### ACKNOWLEDGEMENTS

We would like to thank the clinicians who provided information on eligible cases to the MCTR and the consultant pathologists and haematologists who provided material; Dr T Carr for performing the immunophenotyping; and Mrs E Dale for maintaining the records of the MCTR. We would also like to acknowledge the work of everyone who has contributed towards the MCTR since its inception. The Manchester Children's Tumour Registry is funded by the Cancer Research Campaign. Dr JM Birch is a Cancer Research Campaign Reader in Paediatric Oncology and OB Eden is Cancer Research Campaign Professor of Paediatric Oncology at the University of Manchester.

### REFERENCES

Armstrong AA, Alexander FE, Angus B, Adams J, Cartwright RA, Onions DE and Jarrett RF (1993) Association of Epstein–Barr virus with paediatric Hodgkin's disease. *Am J Pathol* **142**: 1683–1688

© Cancer Research Campaign 1998

Badrinath P, Day NE and Stockton D (1997) Seasonality in the diagnosis of acute lymphocytic leukaemia. *Br J Cancer* **75**: 1711–1713

Birch JM (1988) Manchester Children's Tumour Registry 1954–70 and 1971–83. In *International Incidence of Childhood Cancer*, Parkin DM, Stiller CA, Draper GJ, Bieber CA, Terracini B and Young JL (eds), pp. 299–304, IARC Scientific Publication No. 87. IARC: Lyon

Birch JM and Marsden HB (1987) A classification scheme for childhood cancer. *Int J Cancer* **40**: 620–624

Bjelke E (1964) Leukaemia in children and young adults in Norway. Type, distribution, incidence and survival. *Cancer* **17**: 248–255

Blair V and Birch JM (1994) Patterns and temporal trends in the incidence of malignant disease in children. I. Leukaemia and lymphoma. *Eur J Cancer* **30a**: 1490–1498

Cimino G, Rapanotti MC, Biondi A, Elia L, Lo Coco F, Price C, Rossi V, Rivolta A, Canaani E, Croce C, Mandelli F and Greaves M (1997) Infant acute leukaemia show the same biased distribution of *ALL1* gene breaks as topoisomerase II related secondary acute leukaemias. *Cancer Res* **57**: 2879–2883

Day NE, Smith PG and Lachet B (1985) The latent period of Burkitt's lymphoma: the evidence from epidemiological clustering. In *Burkitt's Lymphoma: a Human Cancer Model*, Lenoir G, O'Conor G and Olweney CLM, (eds), pp. 187–196, IARC Scientific Publication No. 60. IARC: Lyon

Edwards JH (1961) The recognition and estimation of cyclic trends. *Ann Hum Genet* **25**: 83

Forkner CE (1938) *Leukaemia and Allied Disorders*. The Macmillan Company: New York

Fraumeni JF (1963) Seasonal variation in leukaemia incidence. *Br Med J* **2**: 1408–1409

Greaves MF (1988) Speculations on the cause of childhood acute lymphoblastic leukaemia. *Leukaemia* **2**: 120–125

Greaves MF and Alexander FE (1993) An infectious aetiology for common acute lymphoblastic leukaemia in childhood? *Leukaemia* **7**: 349–360

Gunz FW and Spears GFS (1968) Distribution of acute leukaemia in time and space, studies in New Zealand. *Br Med J* **4**: 604–608

Harris RE, Harrell Jr FE, Patil KD and Al-Rashid R (1987) The seasonal risk of paediatric/juvenile acute lymphocytic leukaemia in the United States. *J Chron Dis* **40**: 915–923

Hayes DM (1961) The seasonal incidence of acute leukaemia. A contribution to the epidemiology of the disease. *Cancer* **14**: 1301–1305

Hummel M, Anagnostopoulos I, Dallenbach F, Korbjuhn P, Dimmler C and Stein H (1992) EBV infection patterns in Hodgkin's disease and normal lymphoid tissue: expression and cellular localisation of EBF gene products. *Br J Haematol* **82**: 689–694

Kellett CE (1937) Acute leukaemia in one of identical twins. *Arch Dis Child* **12**: 239–252

Khan G and Coates PJ (1994) The role of Epstein–Barr virus in the pathogenesis of Hodgkin's disease. *J Pathol* **174**: 141–149

Kinlen LJ (1988) Evidence for an infective cause of childhood leukaemia: comparison of a Scottish new town with nuclear reprocessing sites in Britain. *Lancet* **2**: 1323–1327

Kinlen LJ (1995) Epidemiological evidence for an infective basis in childhood leukaemia. *Br J Cancer* **71**: 1–5

Lambin P and Gérard MJ (1934) Variations de fréquence saisonnières de la leucémie aiguë. *Sang* **8**: 730–732

Leck I, Birch JM, Marsden HB and Stewart JK (1976) Methods of classifying and ascertaining children's tumours. *Br J Cancer* **34**: 69–82

Lee JAH (1962) Seasonal variation in clinical onset of leukaemia in young people. *Br Med J* **1**: 1737–1738

Marrero O (1983) The performance of several statistical tests for seasonality in monthly data. *J Statist Comput Simul* **17**: 275–296

Mueller N (1991) An epidemiologist's view of the new molecular biology findings in Hodgkin's disease. *Ann Oncol* **2**: 23–27

Scanu A (1954) Frequenza stagionale delle emopatie acute e sub-acute di tipo leucemico in Campania e in Sardegna (studio statistico). *Riforma med* **68**: 449–452

Walker AM and Van Noord PAH (1982) No seasonality in the diagnosis of acute leukaemia in the United States. *J Natl Cancer Inst* **69**: 1283–1287

Walter SD (1994) Calender effects in the analysis of seasonal data. *Am J Epid* **140**: 649–657

Weinreb M, Day PJR, Green EK, Powell JE, Raafat F, Niggli F and Mann JR (1996) The role of Epstein–Barr virus in Hodgkin's disease from different geographical areas. *Arch Dis Child* **74**: 27–31

Westerbeek RMC, Blair V, Eden OB and Birch JM (1997) The patterns and trends in the incidence of childhood leukaemia and lymphoma (*submitted*)

World Health Organization (1990) *ICD-O: International Classification of Diseases for Oncology*, 2nd edn. World Health Organization: Geneva

© Cancer Research Campaign 1998